# G*ap43* Transcription Modulation in the Adult Brain Depends on Sensory Activity and Synaptic Cooperation

**DOI:** 10.1371/journal.pone.0092624

**Published:** 2014-03-19

**Authors:** Nicole Rosskothen-Kuhl, Robert-Benjamin Illing

**Affiliations:** Neurobiological Research Laboratory, Department of Otorhinolaryngology, University of Freiburg, Freiburg, Germany; Universitat Pompeu Fabra, Spain

## Abstract

Brain development and learning is accompanied by morphological and molecular changes in neurons. The growth associated protein 43 (Gap43), indicator of neurite elongation and synapse formation, is highly expressed during early stages of development. Upon maturation of the brain, Gap43 is down-regulated by most neurons with the exception of subdivisions such as the CA3 region of hippocampus, the lateral superior olive (LSO) and the central inferior colliculus (CIC). Little is known about the regulation of this mRNA in adult brains. We found that the expression of Gap43 mRNA in specific neurons can be modulated by changing sensory activity of the adult brain. Using the central auditory system of rats as a model, Gap43 protein and mRNA levels were determined in LSO and CIC of hearing-experienced rats unilaterally or bilaterally deafened or unilaterally stimulated by a cochlear implant (CI). Our data indicate that Gap43 is a marker useful beyond monitoring neuronal growth and synaptogenesis, reflecting also specific patterns of synaptic activities on specific neurons. Thus, unilateral loss of input to an adult auditory system directly causes asymmetrical expression of Gap43 mRNA between LSOs or CICs on both sides of the brainstem. This consequence can be prevented by simple-patterned stimulation of a dysfunctional ear by way of a CI. We suggest that as a function of input balance and activity pattern, Gap43 mRNA expression changes as cells associate converging afferent signals.

## Introduction

In the auditory system of the adult mammalian brain, FBJ osteosarcoma oncogene *fos* (also known as *c-fos)* is one of the first genes activated by sensory activity evoked either by acoustical or electrical intracochlear stimulation (EIS) [1], [2]. Its protein is a monomer of the heterodimeric Fos-Jun activator protein-1 (AP-1) transcription factor [3]. AP-1 triggers the expression of many genes, among them the growth associated protein Gap43 [4]. This phosphoprotein is neuron-specific and expressed in neuronal somata, axons, and growth cones during pre- and early postnatal development [5], [6]. Gap43 plays a key role during neurite outgrowth in ontogeny and regeneration as well as in early stages of synaptogenesis [7].

In transgenic mice overexpressing *gap43*, aberrant neuronal connections develop [8]. By contrast, complete *gap43* knock-down in mice is lethal in 90% of all cases during the first three weeks of postnatal life [9]. Rekart et al. [10] observed significant memory impairments when Gap43 levels were reduced by 50%. Using Gap43 gene silencing in the olivo-cerebellar system, Gap43 is shown to be essential for maintenance of climbing fiber structure and to promote sprouting after lesion of the inferior olive [11], [12]. Overall, these studies provide evidence that Gap43 is crucial for neuronal network formation.

During postnatal maturation, Gap43 is down-regulated by most neurons and only specific regions of the mammalian brain maintain high levels of Gap43 mRNA [13], [14]. The adult hippocampal CA3 region known to be involved in life-long neuronal plasticity and learning [15], [16] and the lateral superior olive (LSO) as well as the central inferior colliculus (CIC) are notable examples. This suggests that they maintain a responsiveness to reshape their neuronal affiliations upon varying patterns of neuronal activity into adulthood [17], [18]. However, little is known about the factors regulating Gap43 expression in the adult brain.

It has been shown that re-expression of Gap43 protein can be induced by cochlear ablation in the mature auditory brainstem, involving an early phase of low Gap43 levels up to 3 days, and a Gap43 re-expression phase starting after 3 days of deafness with a local maximum of Gap43 protein after 7 days of cochlear ablation [19].

The auditory brainstem nuclei LSO and CIC are indispensable for the binaural computation of sound localization. In order to fulfill this function, LSO neurons receive sensory-driven signals originating from both ears to evaluate interaural level differences (ILD) of acoustic stimuli. Encoding of ILDs depends on excitatory (glutamatergic) input from the cochlear nucleus (CN) and inhibitory (glycinergic) input from the medial nucleus of the trapezoid body (MNTB) of the same side [20]–[25]. Frequency-specific signals arriving from both ears remain tonotopically ordered [26].

The central and major region of the tripartite inferior colliculus (IC) receives tonotopically ordered bilaterally ascending afferents from LSO, lateral lemniscus, CN, and medial superior olive (MSO) [27], and descending input from the auditory cortex and other forebrain regions [28]. Whilst the uncrossed pathways from LSO to CIC are mainly inhibitory, the dominating projection is crossed and excitatory [29].

The utilization of binaural signal processing for spatially directed behavior requires a fine-tuning of neuronal activity depending on converging synaptic inputs. This fine-tuning takes place in postnatal development and needs to be continually readjusted throughout life. Considering the crucial roles of LSO and CIC in binaural signal processing [30], [31], we investigated the effect of experimentally induced deviations in binaural input on *gap43* transcription in their neurons. Gap43 was chosen as an indicator for a neuron’s readiness for activity-dependent structural readjustment of nerve net circuitry. Our hypothesis was that the transcription of *gap43* should change depending on level or pattern of neuronal activity.

## Materials and Methods

### Animals

Twenty nine adult female Wistar rats aged 6 to 16 weeks were used. Care and use of the animals as reported here were approved by the appropriate agency (Regierungspräsidium Freiburg, permission number G-10/83). Rats were anesthetized by an intraperitoneal (i.p.) injection of a mixture of ketamine (50 mg/kg body weight; cp-Pharm Handelsgesellschaft mbH, Burgdorf, Germany) and xylazine (5 mg/kg body weight; Rompun, Bayer-Leverkusen, Germany) before ear bone removal or cochlear opening with electrode insertion. For pain reduction, rats received an intramuscular injection of carprofen (4–5 mg/kg body weight, RIMADYL, Pfizer GmbH, Berlin, Germany). Preceding transcardial perfusion, rats were given a lethal dose of sodium-thiopental (i.p., 250 mg/kg body weight of Trapanal, Nycomed, Konstanz, Germany).

### Experimental Groups

Animals were divided into four experimental groups (Table 1). Guided by previous results [19], [32], we distinguished an ‘early’ phase covering 1 and 3 days after deafness or EIS from a ‘midterm’ phase including 5 and 7 days following deafness or EIS. We investigated an additional ‘late’ phase at 70 days for selected cases (Table 1).

**Table 1:** Experimental Groups.

Group	1 (Co)	2 *(bd)*		3 *(ud)*			4 *(us)*	
treatment	none/malleus removal	bilateral CI implantation		unilateral CI implantation			unilateral CI implantation+EIS	
duration of experiments	–	*early* (1+3 days implanted)	*midterm* (5+7 days implanted)	*early* (1+3 days implanted)	*midterm* (5+7 days implanted)	*late* (70 days implanted)	*early* (1+3 days EIS)	*midterm* (5+7 days EIS)
no. of LSOs	16	4	4	4i/4c	4i/4c	3i/3c	3i/3c	3i/3c
no. of CICs	16	4	4	4i/4c	4i/4c	3i/3c	3i/3c	3i/3c

c: contralateral; i: ipsilateral; Co: control; *bd*: bilateral deaf; *ud*: unilateral deaf; *us:* unilateral stimulation; EIS: electrical intracochlear stimulation.

**Figure 1:**
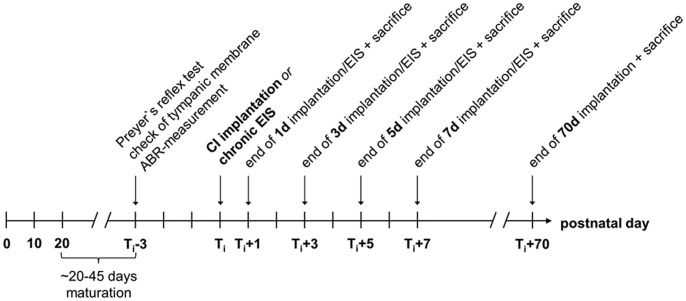
Experimental Design. T_i_: time of implantation of a passive electrode dummy or onset of electrical intracochlear stimulation (EIS); d: day(s).

**Figure 2:**
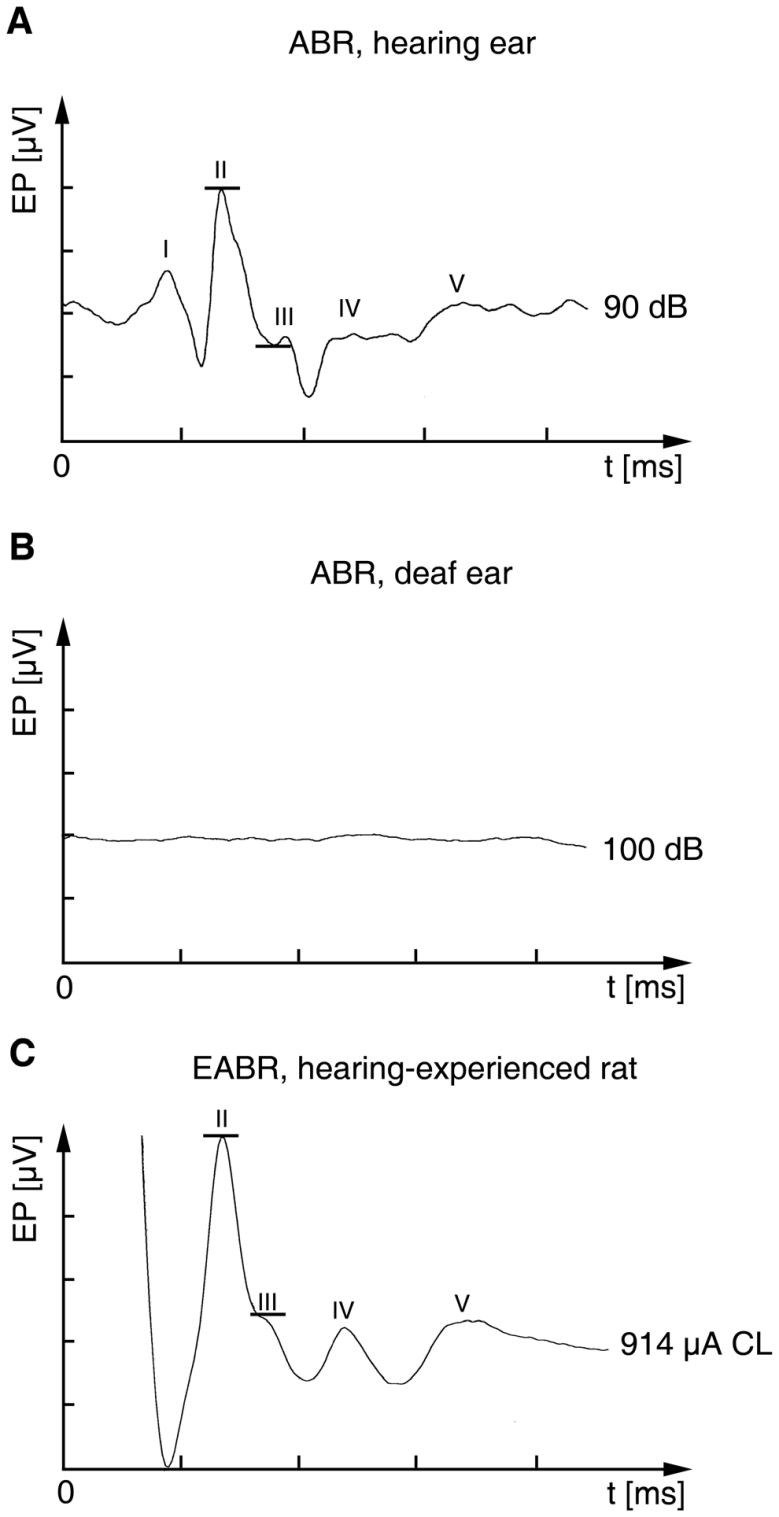
Acoustically and electrically evoked auditory brainstem responses (ABR/EABR). (A, B) Hearing ear stimulated at 90 dB (A) and an ear deafened by electrode insertion stimulated at 100 dB (B). (C) Representative EABR response of a hearing-experienced rat at a current level (CL) of 914 μA. Typically, the ABR of a hearing rat showed five distinguishable peaks (I–V), four of them (II–V) recognizable in the EABR. The black horizontal lines indicate the range of amplitude measurement. x-axis: 2 ms per unit; y-axis: 4 μV per unit; EP: evoked potential.

Group one constitutes age matched controls (Co) without cochlear implant (CI) insertion. To ensure that these control rats had normal hearing, we first tested the motor response to a handclap (Preyer’s reflex [33]) three days before sacrifice (Fig. 1). We then checked tympanic membrane and middle ear under a microscope to verify that hearing is not impaired due to damage of the tympanic membrane or an inflammation of the middle ear. Eventually, we measured the auditory brainstem responses (ABRs) (Fig. 1). For the ABR recording, steel needle electrodes were placed subcutaneously at vertex and mastoids and a 20 Hz train of click stimuli was presented to one side through a brass pipe inserted into the outer ear canal, while the other ear was masked by white noise at the same sound pressure level (SPL). The SPL was stepwise increased, attempting to elicit an ABR at hearing threshold visualized by an averager (Multiliner E; Evolution 1.70c; Toennies & Jäger GmbH, Höchberg, Germany). The ABR mean amplitudes were set after 300 sweeps recorded in a frequency band of 0.1 to 3 kHz (Fig. 2A).

In order to estimate the impact of naturally occurring sound intensities on the transcription of *gap43*, we removed the malleus unilaterally or bilaterally three days before perfusion in five rats leading to a rise of hearing threshold by 50 dB [34]. No statistically significant difference was found against the animals without any intervention by any histochemical measure.

The second experimental group included bilaterally deafened (*bd*) rats (Table 1). Normal hearing was verified by ABR measurement before rats were deafened by bilateral insertion of a CI into the cochlea (Fig. 1). The operation consisted of exposing the cochlea by a retroauricular surgical approach. Preserving the facial nerve, tympanic membrane and malleus were removed and the opening in the bulla tympani was widened to provide good visibility of the cochlea. A 0.6 mm wide hole was made with a diamond drill in the bony wall of the medial turn of the cochlea and two rings of an electrode carrier dummy were inserted without being activated. One and 3 days (early group) as well as 5 and 7 days (midterm group) after electrode insertion, rats were anesthetized for electrode explantation immediately followed by transcardial perfusion (Fig. 1).

The third experimental group consisted of unilaterally deafened (*ud*) rats (Table 1). Unilateral deafness was again induced by CI insertion into the left cochlea, while the right cochlea remained intact. The electrode carrier remained in place for 1 and 3 days, 5 and 7 days, or 70 days. To verify total deafness by CI insertion in experimental groups two and three, we measured the ABRs due to click presentation 1, 3, 5 and/or 7 days after implantation (Fig. 2B).

Finally, experimental group four consisted of unilaterally CI stimulated (*us*) rats (Table 1) as early or midterm group members. Normal hearing capacity was checked before CI stimulation as described for the control group (Fig. 1). The surgical procedure included approach and opening of the cochlea as described above. Before bipolar electrode insertion into the medial turn of the cochlea, the electrode connection device was fixed on the skull with small threaded screws. The electrode carrier was then pulled through under the skin and inserted into the cochlea following the retroauricular surgical approach. To stabilize electrode position, the bulla was filled with 4% lukewarm agar. After hardening, the wound was surgically closed.

### Chronic EIS

**Figure 3:**
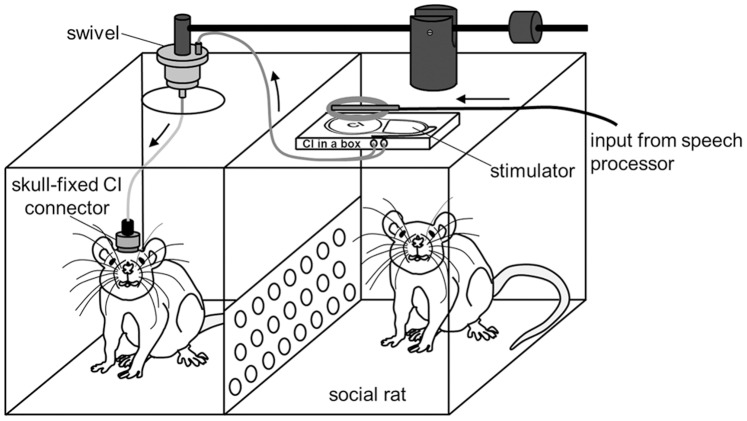
In-house designed setup for chronic electrical intracochlear stimulation (EIS). The rat in the left part of the cage is stimulated using a skull-fixed cochlear-implant (CI) connector. The implanted device was connected to the external stimulator ‘CI in a box’ by way of a swivel. The input for the CI delivers the speech processor. During chronic stimulation, a companion rat resided in the right part of the cage to avoid detrimental effects of social deprivation.

**Figure 8:**
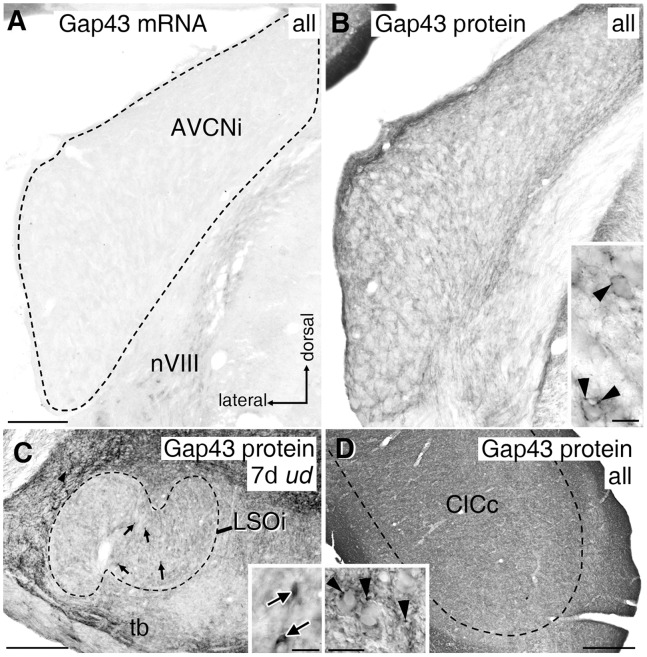
Gap43 mRNA and protein expression in auditory brainstem. (A) Anteroventral cochlear nucleus (AVCN, dashed line) was devoid of Gap43 mRNA staining on both sides under any experimental condition. (B) Faintly stained Gap43 protein-positive axonal boutons were present throughout AVCN in controls and all experimental conditions. Inset: higher magnification of immuno-positive presynaptic endings (arrowheads). (C) Gap43 protein expression in lateral olivocochlear neurons (arrows) within LSOi (dashed line) required at least 5 days (d) of electrode implantation independent of its activation. Inset: close-up of Gap43 protein-positive neurons (arrows) and boutons following 7d of *ud*. (D) Throughout CIC (dashed line), Gap43 immunoreactivity was always present. Inset: close-up of immuno-positive presynaptic endings (arrowheads). Scale bars for A to D: 0.2 mm. Scale bars of insets B to D: 20 μm. LSO: lateral superior olive; CIC: central inferior colliculus; nVIII: 8th cranial nerve; tb: trapezoid body; i: ipsilateral; c: contralateral; *ud*: unilateral deafness.

The implanted device was connected to the external stimulator (CI in a box) by way of a swivel (Fig. 3). The stimulator was connected to a communicator, both kindly provided by Cochlear Germany GmbH & Co. KG. The swivel, directly placed over a centered hole in the cage lid, guaranteed unhindered mobility of the rat during stimulation. Still under anesthesia, the CI was activated to record the electrically evoked auditory brainstem response (EABR; Fig. 2C) with steel needle electrodes placed subcutaneously at vertex and mastoids, respectively. EABR measurement was performed to corroborate correct placement of stimulation electrodes and to determine an appropriate current level. The EABR was visualized using an averager (Multiliner E, Evolution 1.70c, Toennies), calculating mean amplitudes of 500 sweeps in a frequency band of 0.1 to 10 kHz. We aimed to obtain maximal EABR amplitudes of 9 μV±10% by adjusting the current level of EIS to match acoustic stimuli of 90 dB above hearing threshold, causing specific tonotopic activation of central auditory neurons (Figs. 2A, C and 8). After recovering from anesthesia, the CI was activated for causing local activation of the spiral ganglion. Bipolar stimulation consisted of 50 pulses per second biphasic stimuli with a phase width of 50 μs [35]. During chronic stimulation, a companion rat resided in the right part of the cage to avoid detrimental effects of social deprivation (Fig. 3).

### Immunohistochemistry (IHC)

Following completion of postoperative survival time or stimulation period, animals received a lethal dose of sodium-thiopental and were transcardially perfused with a fixative containing 4% paraformaldehyde in 0.1 M phosphate buffer at pH 7.4. After the brain was removed from the skull and soaked in 20% RNase/DNase-free sucrose buffer overnight, parts containing CN, LSO, and CIC were cryo-cut into 30 μm thick frontal sections. Following incubation with 0.045% H_2_O_2_, 1% sodium-borohydride (only for Fos staining) and 1% milk powder, each in 0.02 M sodium phosphate buffer at pH 7.4 for 30 min, sections were exposed to a primary antibody raised in goat against Fos protein (SC-52-G, 1∶2000, lot. no. A2810, Santa Cruz Biotechnology Inc., Santa Cruz, USA), or raised in mouse against Gap43 protein (MAB347, 1∶5000, lot. no. LV178643/NG1894354, Millipore Biosciences, Temecula, USA). After incubation for 48 h at 4°C, a biotinylated secondary anti-goat (BA-5000, 1∶200, Vector Laboratories, Inc., Burlingame, USA) or anti-mouse antibody (BA-2001, 1∶200, Vector Laboratories) was added. Visualization of anti-Fos (goat) and anti-Gap43 (mouse)-binding sites was based on intensification by the avidin-biotin-technique (Vector Laboratories), followed by 3,3′ diaminobenzidine tetrahydrochloride (Sigma, Taufkirchen, Germany). Controls omitting primary antibodies were run to verify their specificity and lack of unspecific binding by the secondary antibody. Neuronal nuclei of the parabrachial region always containing Fos immunoreactivity served as positive staining controls [36].

### Synthesis of Fos and Gap43 Fragment for in-situ Hybridization (ISH)

Gap43 mRNA was isolated (PAXgene RNA Kit 762174 preAnalytiX, QIAGEN, Hilden, Germany) from rat CN at postnatal day 10, while for Fos fragment synthesis mRNA was isolated from adult rat CN after 2 h EIS. Afterwards, subsequent complementary DNA (cDNA) synthesis was performed using standard techniques (Omniscript RT Kit 205111, QIAGEN).

#### Gap43 DNA fragment amplification

Based on the cDNA, a specific DNA fragment (191 bp) of the Gap43 gene [NCBI: NM_017195.3, GI: 166091453] flanked with the T3 and T7 promoters was amplified with the following primers: Gap43 F4 (T3)

(5′-ATTAACCCTCACTAAAGGGATGCAGAAAGCAGCCAAGCTGAGGA-3′), and Gap43 R3 (T7)

(5′-GGGCAACGTGGAAAGCCGTTTCTTGGGATATCACTCAGCATAAT-3′).

#### Fos DNA fragment amplification

Based on the cDNA, a specific DNA fragment (313 bp) of the Fos gene [NCBI: NM_022197, GI: 148298807] was amplified with the following primers: F1-FBJ/Fos (5′-AGCTCCCACCAGTGTCTACC-3′), and R1-JB/Fos 5′-CCACGGAGGAGACCAGAGTG-3′.).

#### Riboprobe construction

**Figure 4:**
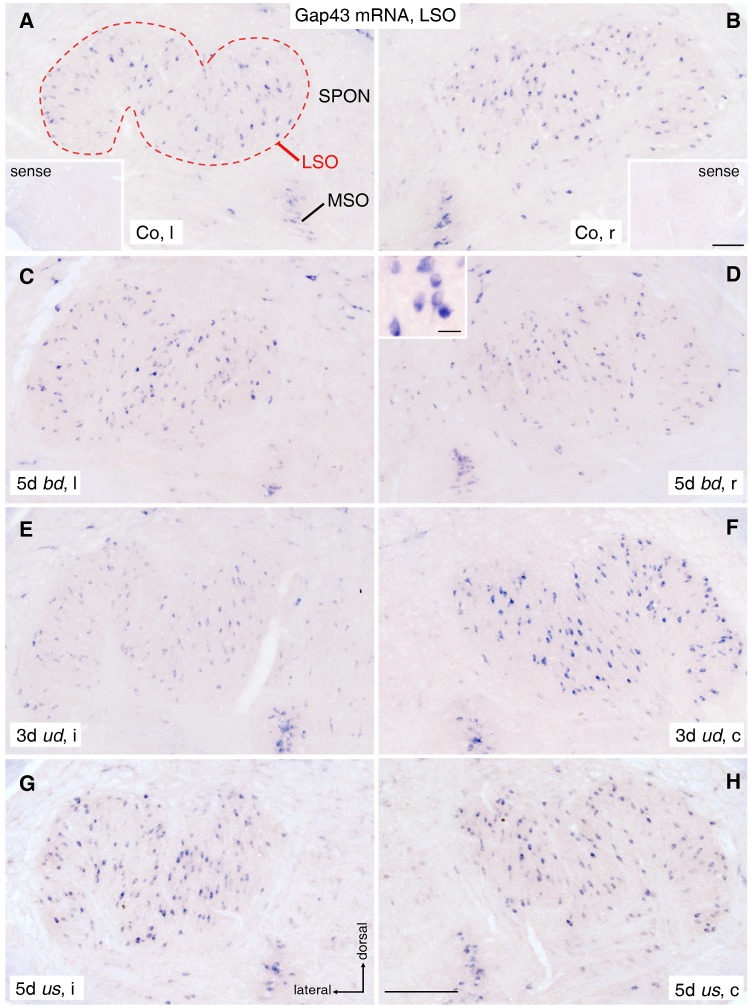
Gap43 mRNA staining in lateral superior olive (LSO). (A, B) A basal level of Gap43 mRNA was seen in neuronal cell bodies (purple dots) of the entire left (A) and right (B) LSO (dashed outline) of untreated control rats (Co). The insets show lack of staining in corresponding sections after use of Gap43 sense probe. Scale bar for insets: 0.2 mm. (C, D) Gap43 mRNA expression in left (C) and right (D) LSO after 5 days (d) of bilaterally deaf (*bd*) rats is equivalent to control level. Inset in D shows Gap43 mRNA positive neurons at higher magnification. Scale bar of inset: 20 μm. (E) Following 3d of unilateral deafness (*ud*), Gap43 mRNA expression decreased in neurons of LSOi compared to controls. (F) Simultaneously, the expression increased contralaterally. (G, H) After 5d of unilateral stimulation (*us*), high bilaterally balanced Gap43 mRNA levels were observed. Scale bar for A to H: 0.2 mm. SPON: superior paraolivary nucleus; MSO: medial superior olive; l: left; r: right; i: ipsilateral; c: contralateral.

**Figure 5:**
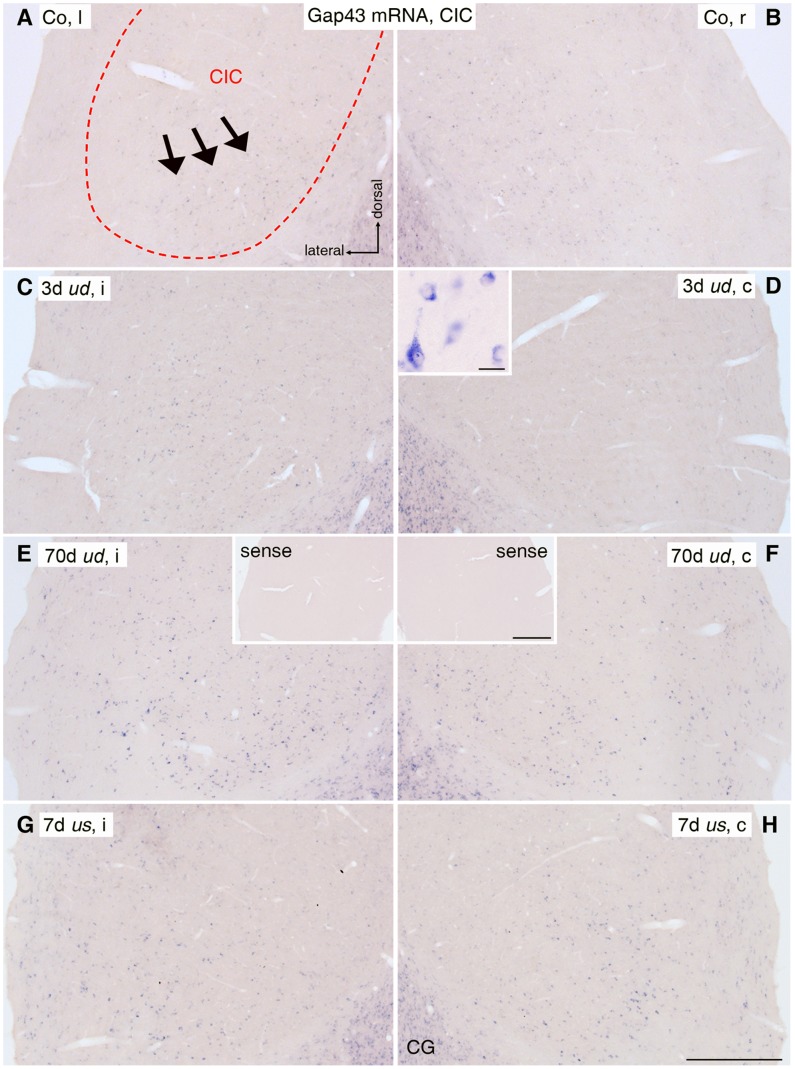
Gap43 mRNA staining in central inferior colliculus (CIC). (A, B) In the untreated control group (Co), Gap43 mRNA positive neurons (purple dots) in CIC (dashed outline) were mainly localized ventrally (arrows). (C, D) 3 days (d) after unilateral deafness *(ud*), Gap43 mRNA level increased in CICi (C), while expression was decreased in CICc (D). The inset shows a higher magnification of stained CIC neurons. Scale bar in inset: 20 μm. (E, F) After 70d of *ud*, Gap43 mRNA expression rose bilaterally in CIC, with a significantly higher level on the ipsilateral side (E) compared to the contralateral side (F). Insets show control staining after incubation with Gap43 sense probe. Scale bar for insets: 0.2 mm. (G, H) Unilateral stimulation (*us*) for 7d resulted in an increase of Gap43 mRNA levels in ventral CIC on ipsilateral (G) and contralateral (H) side. Scale bar for A to H: 0.2 mm. l: left; r: right; i: ipsilateral; c: contralateral; CG: central gray.

The Fos fragment was subcloned into pCR4-TOPO (K4595-01, Invitrogen, Carlsbad, USA) to construct subclone pCR4 Fos. From this construct a linearized Fos fragment was amplified, which is flanked by the T3 and T7 promoters. For both fragments, digoxigenin-labeled antisense riboprobes were generated, from the 191 bp rat Gap43 cDNA and the 313 bp rat Fos cDNA after transcription with T7 (for Gap43) or T3 (for Fos) RNA polymerase (for Gap43: Cat. No. 10881767001; for Fos: Cat. No. 11031163001, Roche Applied Science, Mannheim, Germany). Sense riboprobes were made after transcription with T3 (for Gap43) or T7 (for Fos) RNA polymerase (for Gap43: Cat. No. 11031163001, for Fos: Cat. No. 10881767001, Roche Applied Science). These sense probes served as control to verify that the complementary transcript failed to generate any staining in the entire CN, LSO (cp. Fig. 4, inset in A, B), and CIC, (cp. Fig. 5, inset in E, F).

### In-situ Hybridization (ISH)

Thirty micrometer thick cryo-cut frontal brain sections were collected in 2× standard saline citrate (SSC) buffer (Invitrogen, Life Technologies GmbH, Darmstadt, Germany). Sections were washed in 2× SSC buffer for 15 min. Before pre-hybridization, they were pretreated in a 1∶1 dilution of 2× SSC and a hybridization buffer consisting of 50% formamide (Carl Roth GmbH, Karlsruhe, Germany), 4× SSC (Invitrogen), 10% dextransulfate (Sigma), 1× Denhardt’s solution (AMRESCO Inc., Solon, USA), 250 μg/ml heat-denatured cod and herring sperm DNA (Roche Diagnostics GmbH, Mannheim, Germany), and 625 μg/ml tRNA from E. coli MRE 600 (Roche) for 15 min. Pre-hybridization in hybridization buffer at 55°C lasted for 60 min. Hybridization was done overnight at 55°C in the same solution with the addition of 100 ng/ml digoxigenin (DIG)-labeled Fos and 1000 ng/ml DIG-labeled Gap43 antisense or sense complementary RNA, respectively. After hybridization, sections were washed twice in 2× SSC for 15 min at room temperature, 2× SSC and 50% formamide (MERCK KGAA, Darmstadt, Germany) for 15 min, 0.1× SSC and 50% formamide for 15 min, and twice in 0.1× SSC for 15 min at 65°C each. For immunological detection of DIG-labeled hybrids, brain sections were treated twice in buffer 1 (100 mM Tris/HCl, pH 7.5; 150 mM NaCl) for 10 min each, blocked in buffer 2 (1% blocking reagent [Roche] in buffer 1) for 60 min at room temperature, and incubated overnight at 4°C with the anti-DIG Fab fragment from sheep tagged with alkaline phosphatase (1∶1500, Roche) in buffer 2. For the preparation of color reaction, sections were equilibrated in buffer 1 for 2× 10 min each and in buffer 3 (100 mM Tris/HCl, pH 9.5; 100 mM NaCl; 50 mM MgCl_2_) for 10 min. Afterwards, nitroblue tetrazolium (0.34 mg/ml, Roche) and 5-bromo-4-chloro-3-indolyl-phosphate, 4-toluidine salt (0.17 mg/ml, Roche) were added to buffer 3. Development of the color reaction was performed in the dark at room temperature, eight hours for Gap43 mRNA staining, and 9 h for Fos mRNA staining. The color reaction was stopped by transfer sections into distilled water. Finally, sections were mounted on glass slides, dehydrated in increasing grades of alcohol, cleared in xylene, and coverslipped with DPX (Sigma-Aldrich, Steinheim, Germany).

### Detection and Quantification of Gap43 mRNA Staining Intensity

To quantify changes in Gap43 mRNA expression, the mean of staining intensities for Gap43 mRNA were detected throughout LSO and CIC on both sides of the brainstem. Color photographs were taken from sections of the left (l)/ipsilateral (i) and right (r)/contralateral (c) side of mRNA stained LSOs and CICs through a x10 (for LSO) or a x5 (for CIC) objective with a digital camera (AxioCam, Zeiss, Jena, Germany) at 8 bit conversion. Under graphics software (Adobe Photoshop CS, Adobe Systems Inc., San Jose, USA) global variations in staining intensity of sections from different animals were compensated by setting the median of each photograph to 217 (for LSO) or 185 (for CIC). We then extracted the red channel of each photograph. Selection of a region of interest (ROI) was done using the lasso tool (Figs. 4A, 5A, dashed lines). For LSO, the ROI was defined by tracing the histologically visible border of each LSO, whereas the same defined ROI was always used for CIC of different rats. Detection of the mean staining intensity of Gap43 mRNA positive neurons in LSO and CIC was performed inside ROIs by using the filling tool at tolerance 10 (for LSO) or 11 (for CIC). Due to this definition of staining intensity, LSO and CIC lacking Gap43 mRNA positive neurons had a mean value of 0, whereas a maximally stained single neuron has a staining intensity of around140 (for LSO) or 170 (for CIC). Due to the looser packing of neurons and the restriction of positive neurons to only a part of the ROI, mean values of staining intensity per ROI detected in this study varied between 2 and 14 for LSO, and between 0.5 and 1.7 for CIC, across all types of experiments.

### Statistical Analysis

**Figure 6:**
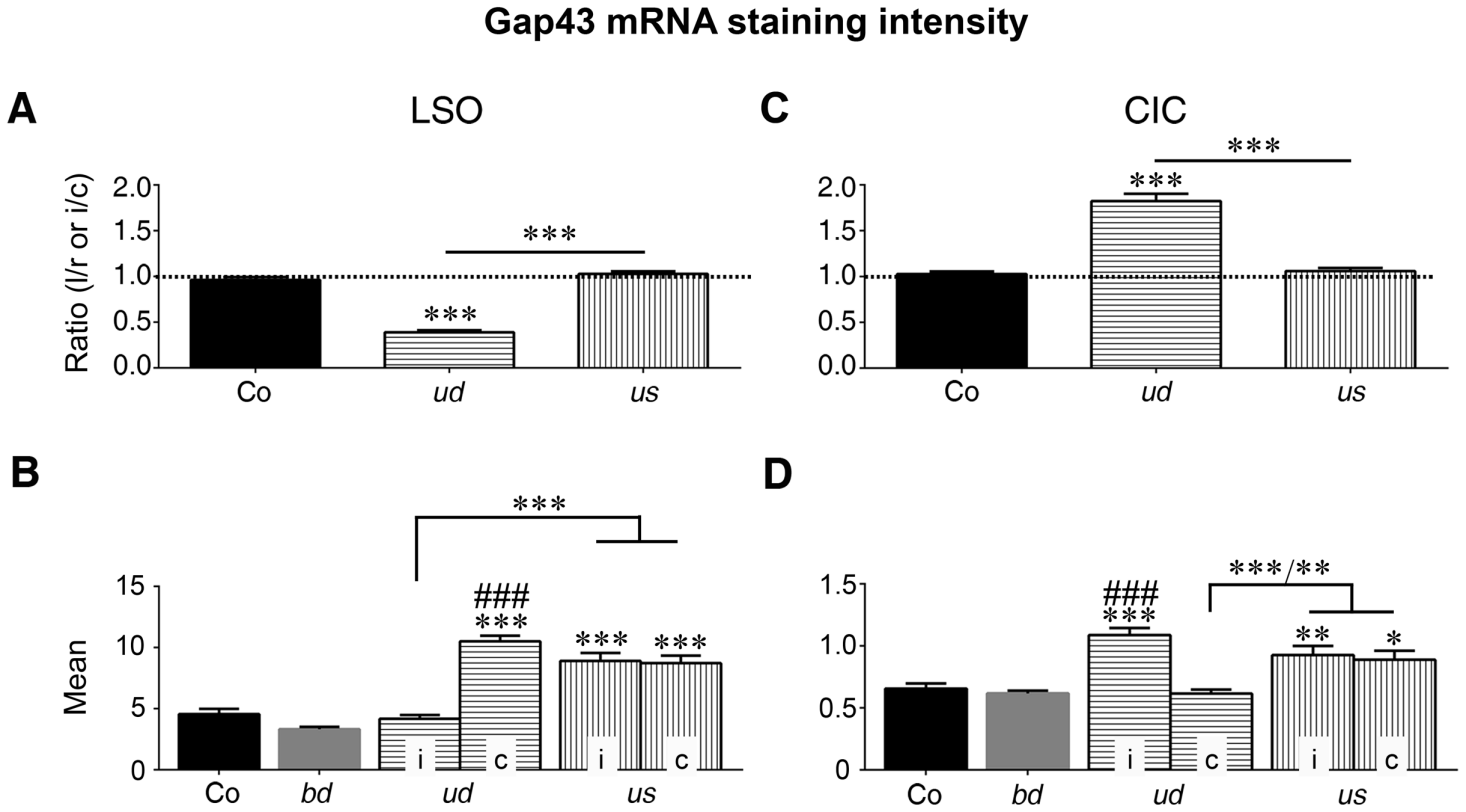
Quantification of Gap43 mRNA staining intensities in LSO (A, B) and CIC (C, D). Bilateral comparison of left-to-right or ipsilateral-to-contralateral staining intensities in LSO (A) and CIC (C) indicates that Gap43 mRNA was different between both sides of the brainstem if sensory stimulation was reduced on one side only (LSO: n = 19/92 rats/slices; F = 45.48, DFn = 2, DFd = 118; *ud*: 0.39±0.02; Co: 0.96±0.03; CIC: n = 19/89 rats/slices; F = 201.5, DFn = 2, DFd = 116; *ud*: 1.82±0.08; Co: 1.03±0.03; p<0.001 for both). EIS in the ear of one side with the other ear continuing transduction of acoustically signals maintained ipsilateral-to-contralateral balance as in controls (LSO: n = 6/29 rats/slices; *us*: 1.03±0.03; CIC: n = 6/30 rats/slices, *us*: 1.06±0.03; p>0.05 for both). Dotted line indicates bilateral symmetry (1.0). Significant differences against control level are indicated by asterisks above bars. Significant divergences between *ud* and *us* rats are indicated by lines with associated asterisks. (B) Staining results of *gap43* transcription in LSO (n = 29/142 rats/slices; F = 53.89, DFn = 5, DFd = 278) indicates that the staining intensity increased significantly against controls (Co) in LSOc due to unilateral deafness (*ud*; n = 19/89 rats/slices; p<0.001), and in bilateral LSO after unilateral stimulation (*us*; n = 14/55 rats/slices; p<0.001 for both). Additionally, staining intensity in *ud* rats on the contralateral side was significantly higher than for bilaterally deafened (*bd*) rats (n = 15/87 rats/slices; ###: p<0.001). Staining intensities of both LSOs of *us* rats were higher than the ipsilateral intensity of *ud* rats (n = 17/90 rats/slices; p<0.001 for both). (D) Staining results of *gap43* transcription in CIC (n = 29/145 rats/slices; F = 18.71, DFn = 5, DFd = 284) revealed that the level increased against controls in CICi due to *ud* (n = 19/91 rats/slices; p<0.001), and in bilateral CIC after *us* (n = 14/56 rats/slices; p = 0.0085 for Co vs. *us* i; p = 0.0385 for Co vs. *us* c). Mean staining intensity of CIC of *bd* rats was significantly different from CICi of *ud* rats (n = 15/89 rats/slices; ###: p<0.001), whereas both sides of *us* rats rose against CICc of *ud* rats (n = 17/95 rats/slices; p = 0.0007 for *us* i vs. *ud* c; p = 0.0049 for *us* c vs. *ud* c). Significance levels: (***/^###^) for p<0.001, (**) for p<0.01, (*) for p<0.05. LSO: lateral superior olive; CIC: central inferior colliculus; i: ipsilateral; c: contralateral.

**Figure 7:**
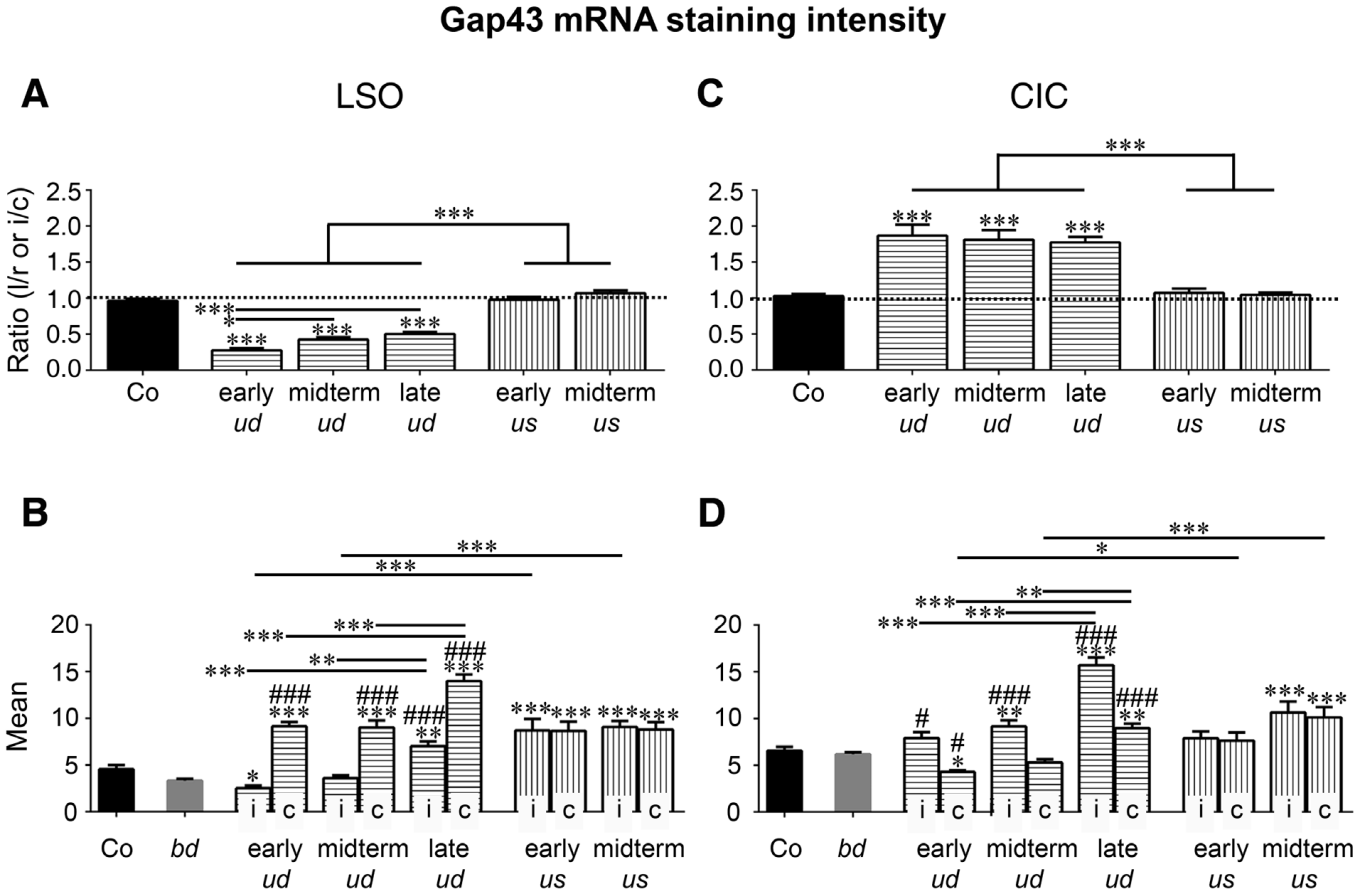
Quantification of Gap43 mRNA staining intensities in LSO (A, B) and CIC (C, D) in experimental subgroups. (A, C) Bilateral comparison of left-to-right or ipsilateral-to-contralateral staining intensity of LSO (A) and CIC (C) indicates that Gap43 mRNA was significantly different between both sides of the brainstem if sensory stimulation was reduced on one side only (*ud)* compared to control (LSO: n = 19/92 rats/slices; F = 102.5, DFn = 5, DFd = 115; early *ud*: 0.28±0.03; midterm *ud*: 0.42±0.04; late *ud*: 0.50±0.03; Co: 0.96±0.03; CIC: n = 19/89 rats/slices; F = 19.34, DFn = 5, DFd = 119; early *ud*: 1.87±0.15; midterm *ud*: 1.81±0.13; late *ud*: 1.77±0.08; Co: 1.03±0.03; p<0.001 for all). Comparison of the ratios of the three subgroups of *ud* rats showed significant differences among temporal groups only for LSO (p = 0.0208 for early *ud* vs. midterm *ud*; p<0.001 for early *ud* vs. late *ud*). Activating a cochlear implant on one ear (*us*) with the other ear hearing acoustically maintained an ipsilateral-to-contralateral balance (LSO: n = 6/29 rats/slices; early *us*: 0.98±0.04; midterm *us*: 1.07±0.04; CIC: n = 6/30 rats/slices, early *us*: 1.07±0.06; midterm *us*: 1.05±0.03; p>0.05 for all). For both LSO and CIC, significant differences were detected between the subgroups of *ud* and *us* rats (p<0.001 for all). Dotted line indicated bilateral symmetry (1.0). Significant differences against control level were indicated by asterisks above bars. (B) Results for *gap43* transcription in LSO (n = 29/142 rats/slices) indicated that staining intensity increased significantly against controls (Co) in LSOc of *ud* rats independent of the duration of deafness (n = 19/89 rats/slices; F = 37.55, DFn = 11, DFd = 272; p<0.001). By contrast, the mean staining intensity decreased after early *ud* in LSOi (p = 0.0158), while it increased significantly after late *ud,* compared to controls (p = 0.0069). Additionally, the staining intensity of all *ud* rats on the contralateral side was significantly higher as compared to bilaterally deafened (*bd*) rats (n = 15/87 rats/slices; F = 86.54, DFn = 6, DFd = 167; ###: p<0.001). On the ipsilateral side, a significant rise against the staining intensity of *bd* was verified only following a longer duration of *ud* (###: p<0.001). A comparison of late *ud* rats with early and midterm *ud* rats showed a bilateral increase of mean Gap43 mRNA staining intensity with sustained duration of deafness (F = 27.52, DFn = 9, DFd = 170; p = 0.002 for midterm *ud* i vs. late *ud* i; p<0.001 for all other). After *us,* neurons in both LSOs increased their *gap43* transcription level independent of stimulation duration against controls (F = 37.55, DFn = 11, DFd = 272; p<0.001 for all). Staining intensities of LSOi of both subgroups of *us* rats were higher than the ipsilateral intensities of isochronously *ud* rats (n = 17/90 rats/slices; F = 27.52, DFn = 9, DFd = 170; p<0.001 for both). (D) Results for *gap43* transcription in CIC (n = 29/145 rats/slices) showed that staining intensity increased against controls in CICi of *ud* rats after a midterm and late period of deafness (n = 19/91 rats/slices; F = 25.16, DFn = 11, DFd = 278; p = 0.0028 for midterm ud i vs. Co; p<0.001 for late *ud* i vs. Co). By contrast, the mean staining intensity decreased after early *ud* in CICc (p = 0.0179), while it increased significantly after late *ud* compared to controls (p = 0.0094). Staining intensities of all *ud* rats on the ipsilateral side were significantly higher than for bilaterally deafened (*bd*) rats (n = 15/89 rats/slices; F = 57.93, DFn = 6, DFd = 171; #: p = 0.03 for early *ud* i vs. *bd*; ###: p<0.001 for other both). Contralaterally, a significant rise against the staining intensity of *bd* was seen only following longer lasting *ud* (###: p<0.001), while it significantly decreased after early *ud* (#: p = 0.01). A comparison of late *ud* rats with early and midterm *ud* rats showed a bilateral increase of mean Gap43 mRNA staining intensities with duration of deafness (F = 22.22, DFn = 9, DFd = 180; p = 0.001 for midterm *ud* c vs. late *ud* c; p<0.001 for all other). After *us,* both CICs increased their *gap43* transcription level significantly only after a midterm duration of *us* compared to control (25.16, DFn = 11, DFd = 278; p<0.001 for both). Staining intensities of CICc of both subgroups of *us* rats were higher than the contralateral intensities of isochronously *ud* rats (n = 17/95 rats/slices; F = 22.22, DFn = 9, DFd = 180; p = 0.0155 for early *us* c vs. early *ud* c; p<0.001 for midterm *us* c vs. midterm *ud* c). Significance levels: (***/^###^) for p<0.001, (**) for p<0.01, (*/^#^) for p<0.05. LSO: lateral superior olive; CIC: central inferior colliculus; i: ipsilateral; c: contralateral.

Statistical analysis was done with Prism (GraphPad Software, La Jolla, USA) and Microsoft Office Excel 2003 (Microsoft Germany GmbH, Unterschleiβheim, Germany). Mean staining intensities and their standard error (SEM) for Gap43 mRNA were determined for LSO and CIC in twenty nine brains of three to seven pairs of sections (Figs. 4A, 5A, dashed line). Across the respective auditory area on each side, ratios of left-to-right (controls and *bd* rats) or ipsilateral-to-contralateral (*ud* and *us* rats) sides were calculated for subsequent statistical analysis (Figs. 6, 7).

Differences of means were identified by one-way analysis of variance (ANOVA) with significance level set to p<0.05. The F-values of each ANOVA test with their degrees of freedom (DF) are specified in the legends of Figs. 6 and 7. Supplemented by Dunnett post-hoc test, significant differences of means of experimental groups with the single control group were determined. To determine means that are significantly different from each other within a defined subset, Tukey post-hoc test was added. Significant differences of means for selected pairings of groups were calculated by combining one-way ANOVA with Sidak post-hoc test. Again, the significance level was set to p<0.05. Significance levels were distinguished as (***/^###^) for p<0.001, (**) for p<0.01, (*/^#^) for p<0.05. To detect differences between left and right brainstem side of controls, a two-tailed unpaired t-test was used with confidence interval set to p<0.05.

Preparatory statistics of three different control groups: non-operated control rats (I) as well as rats with unilateral (II) or bilateral (III) malleus removal, revealed that Gap43 mRNA expression was indistinguishable among them. Specifically, no statistical differences were detected between ipsilateral-to-contralateral ratios of *gap43* transcription of the three different groups (n = 8/30 rats/slices; for LSO: (I): 1.02±0.028, mean ± SEM; (II): 0.99±0.06; (III): 0.89±0.06; for CIC: (I): 0.99±0.032; (II): 1.14±0.03; (III): 1.01±0.06; p>0.05 for all), leading to a pooling of all control rats to one group (see ‘Experimental groups’) for the following statistic of this study.

Gap43 staining levels were evaluated for bilaterally deafened (*bd*) rats. Staining levels were indistinguishable between both sides of LSO and of CIC, respectively, as well as between the two time windows studied (LSO: 4/25 rats/slices; early *bd* l: 2.94±0.34; early *bd* r: 3.32±0.37; midterm *bd* l: 3.63±0.40; midterm *bd* r: 3.49±0.53; CIC: 4/24 rats/slices; early *bd* l: 0.59±0.042; early *bd* r: 0.62±0.04; midterm *bd* l: 0.62±0.045; midterm *bd* r: 0.63±0.05; p>0.05 for all). Consequently, we combined the Gap43 mRNA staining intensities of LSOs or CICs to one group for further statistical analysis.

## Results

### Intracochlear Electrode Insertion Induces Total Deafness

Bilaterally opening the cochleae and inserting electrodes into their medial turn consistently disabled implanted rats to show a motor response to a handclap (Preyer’s reflex). This indicates a rise of ABR threshold beyond 81 dB SPL [33]. Audiometry revealed a rise of hearing threshold by 100 dB (Fig. 2B) against normal hearing controls (Fig. 2A), implying total deafness. Correspondingly, unilateral electrode implantation caused unilateral deafness.

### Neurons of the Mature Auditory Brainstem Maintain *gap43* Transcription

Under control conditions, neurons throughout LSO (Fig. 4A, B) and in the ventral part of CIC (Fig. 5A, B) possessed a notable level of Gap43 mRNA in their cytoplasm (Figs. 4D, 5D inset), showing a mean staining intensity of 4.56±0.42 for LSO (Fig. 6B) and 0.66±0.041 for CIC (Fig. 6D), both against 0 for the mean staining level obtained by using the sense probe (Figs. 4A, B, 5E, F insets). Because of a staining ratio close to 1 (for LSO: 0.96±0.03; for CIC: 1.03±0.03; p>0.05 for left vs. right by t-test; Fig. 6A, C), brains were proved to be symmetrical and control data were pooled from both sides of the brainstem.

Our experiments demonstrated that even if sensory-evoked activity fails due to bilateral hearing loss, the basal transcription of *gap43* was unaffected in neurons of LSO and CIC (cp. 4C, D). Statistical evaluation of the mean Gap43 mRNA staining intensity of LSO and CIC revealed no significant difference compared to the staining intensity of control rats (for LSO: *bd*: 3.32±0.2; Co: 4.56±0.42; for CIC: *bd*: 0.62±0.66; Co: 0.66±0.04; p>0.05 for both; Fig. 6B, D).

### Asymmetric Sensory Activity Results in Imbalanced *gap43* Transcription. LSO

Already by 1 day after *ud*, a significant imbalance of Gap43 mRNA staining intensity was found between ipsilateral (i) and contralateral (c) LSO (Fig. 4E, F), with an ipsilateral-to-contralateral ratio of staining intensity significantly smaller than under control conditions (n = 19/92 rats/slices; *ud*: 0.39±0.02; Co: 0.96±0.03; p<0.001; cp. Fig. 6A). For the generation of Fig. 6, data of early, midterm, and late *ud* rats were pooled (cp. Fig. 7). This imbalance resulted from an increase of Gap43 mRNA staining intensity on the contralateral side (*ud* c: 10.51±0.46; p<0.001; Fig. 6B), while the ipsilateral side remained at control level (*ud* i: 4.17±0.32; Co: 4.56±0.42; p>0.05; Figs. 6B, 7B). A quantitative evaluation of randomly selected sets of LSOs from Co, *ud* and *us* rats revealed that these changes in staining levels were not due to varying numbers of stained neurons (n = 9/34 rats/slices; F = 1.712, DFn = 2, DFd = 31; p>0.05; Fig. 4).

In order to see if a once unbalanced auditory system has the potential to re-balance itself after a longer period of deafness, Gap43 mRNA staining intensity was studied after 70 days of *ud*. Still, we detected a significant imbalance of Gap43 mRNA staining between both sides of LSO indicated by a ratio significantly smaller than for controls (late *ud*: 0.50±0.03; Co: 0.96±0.03; p<0.001; Fig. 7A).

### CIC

Within 1 day of *ud*, a sustained imbalance of Gap43 mRNA staining intensity developed between ipsilateral and contralateral CIC (Fig. 5C, D). The ipsilateral-to-contralateral ratio of staining intensity was significantly larger than for controls (n = 19/89 rats/slices; Co: 1.03±0.03; *ud*: 1.82±0.08; p<0.001; Figs. 6C, 7C). Unlike the situation in LSO, this imbalance resulted from a significant increase of Gap43 mRNA staining intensity on the ipsilateral side (*ud* i: 1.09±0.06; p<0.001; Fig. 6D), while the contralateral side remained at control level (*ud* c: 0.62±0.03; Co: 0.66±0.04; p>0.05; Figs. 6D, 7D).

As described for LSO, CIC was still out of balance after 70 days of *ud* (Fig. 5E, F). By this time, an ipsilateral-to-contralateral staining ratio indistinguishable from early and midterm *ud* rats (early: 1.87±0.15; midterm: 1.81±0.13; late *ud*: 1.77±0.08; p>0.05; Fig. 7C) was seen. This led *us* to pool all three groups for statistical evaluation as shown in Fig. 6C, D.

### Unilateral EIS Results in Balanced *gap43* Transcription in Auditory Brainstem Regions. LSO

Within the group of *us* rats, neurons throughout both LSOs expressed balanced levels of Gap43 mRNA independent of stimulation duration (Fig. 4G, H). As a result, the ipsilateral-to-contralateral staining ratio for Gap43 mRNA was found to be close to 1 and thus indistinguishable from controls (n = 14/55 rats/slices; *us*: 1.03±0.03; Co: 0.96±0.03; p>0.05; Figs. 6A, 7A). Compared to the corresponding ratio in *ud* rats, a significant difference was detected (n = 17/91 rats/slices; *us*: 1.03±0.03; *ud*: 0.39±0.02; p<0.001; Figs. 6A, 7A). Unlike controls and LSOi of *ud* rats, the mean staining intensity of Gap43 mRNA positive neurons was bilaterally increased for all stimulation times (n = 25/117 rats/slices; *us* i: 8.91±0.66; *us* c: 8.72±0.62; *ud* i: 4.17±0.32; Co: 4.56±0.42; p<0.001 for all; Figs. 6B, 7B).

### CIC

As described for LSO, stimulation of one ear by a CI resulted in a bilaterally balanced transcription of *gap43* within the cytoplasm of neurons located ventrally in CIC (Fig. 5G, H). Evaluation of the ipsilateral-to-contralateral staining intensity revealed a ratio close to 1 for all different stimulation periods (Figs. 6C, 7C) and similar to controls (n = 14/60 rats/slices; *us*: 1.06±0.03; Co: 1.03±0.03; p>0.05), but significantly different from *ud* rats (n = 17/89 rats/slices; *us*: 1.06±0.03; *ud*: 1.82±0.08; p<0.001; Fig. 6C). However, the mean Gap43 mRNA staining intensities in *us* rats were bilaterally increased compared to controls as well as to CICc of *ud* rats (n = 25/121 rats/slices; *us* i: 0.93±0.07; *us* c: 0.89±0.07; *ud* c: 0.62±0.03; Co: 0.66±0.04; p = 0.0385 for Co vs. *us* c; p = 0.0085 for Co vs. *us* i; p = 0.0049 for *ud* c vs. *us* c; p = 0.0007 for *ud* c vs. *us* i; Fig. 6D). By contrast to the increase in Gap43 staining intensities in LSO, the increase in CIC was mainly based on a substantial rise of *gap43* transcription after the early period of *us* (Fig. 7D).

### Adult *gap43* Transcription in Other Auditory Brainstem Regions

The ventral cochlear nucleus (VCN) of rats remained virtually free of any hybridization under all conditions (cp. Fig. 8A). Apart from LSO and CIC, Gap43 mRNA staining was prominent in neurons of MSO and still noticeable in shell neurons of LSO and in neurons of various other subnuclei of the superior olivary complex. As staining in all of these regions remained unaffected by any experimental condition (Fig. 4), they were not considered any further in this study.

### Gap43 Protein Expression in the Adult Auditory Brainstem

Immunohistochemistry for Gap43 protein in VCN of normal adult rats revealed faintly labeled immunopositive boutons previously shown to be presynaptic endings [37] across the entire nucleus (cp. Fig. 8B and arrowheads in inset). This basal level of specific staining persisted independent of experimental treatment (cp. Fig. 8B) and was generally more prominent than we reported in our earlier work [19], [37], for which tissue fixation including glutaraldehyde was used.

In LSO, no obvious changes in Gap43 protein expression were observed despite prominent changes in Gap43 mRNA level in the cytoplasm of neurons. Only isolated cells contained Gap43 protein on the deaf or stimulated side (Fig. 8C, arrows and inset). However, development of Gap43 protein within these neuronal somata, likely to be LOC neurons [38], required at least 5 days of deafness or chronic EIS. A basal level of immunopositive fibers and presynaptic endings was observed in LSO, with the highest density consistently found in its medial region. These Gap43 immunoreactive endings appeared to remain unaltered on both sides of LSO in all four experimental groups (cp. Fig. 8C).

On the level of CIC, Gap43 immunoreactivity was notable in an area-wide network of stained thin fibers and small boutons which we identified as presynaptic endings in an ultrastructural investigation (unpublished results) (Fig. 8D, and arrowheads in inset). As described for LSO, immunoreactivity was found to be unaffected by experimental treatment on both sides of the brainstem.

### Expression of Fos mRNA and Protein Decreases with Increasing Stimulation Time

**Figure 9:**
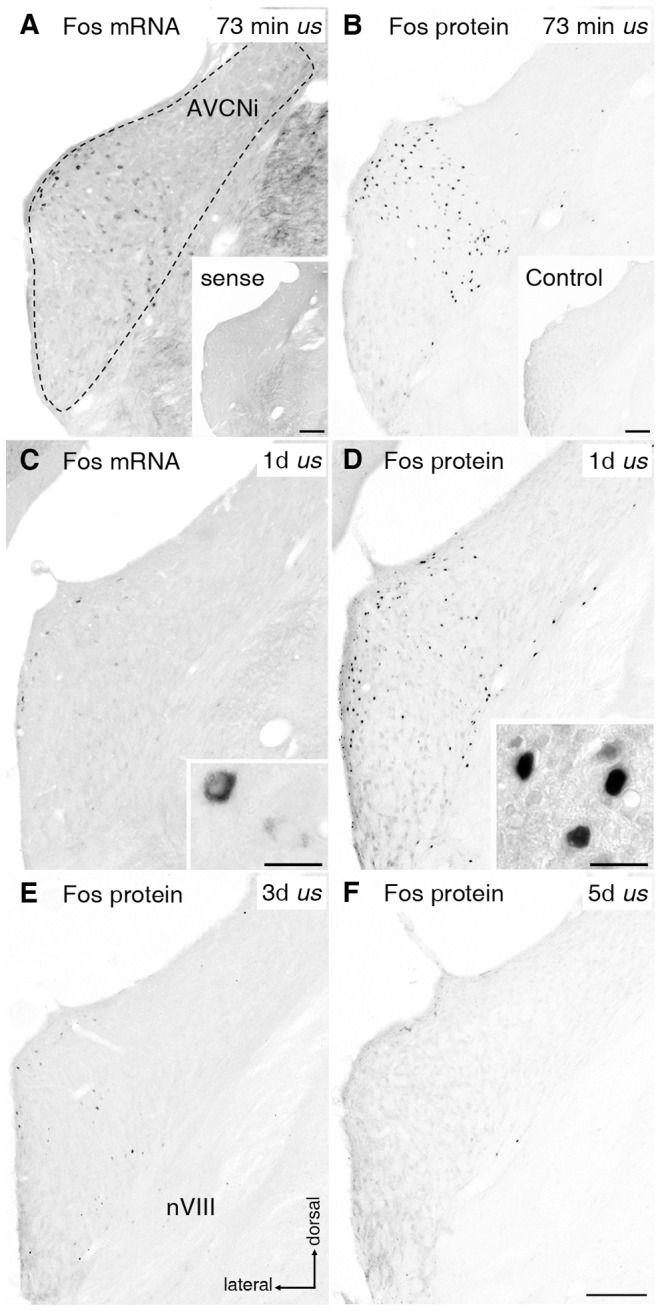
The effectiveness of electrical intracochlear stimulation (EIS) on neurons of the anteroventral cochlear nucleus (AVCN). Fos mRNA (A) and Fos protein (B) closely corresponded locally after EIS for under 2 h duration. (A, C) Fos mRNA expression (black dots) in neurons of ipsilateral (i) AVCN after 73 min (A; cp. Illing and Rosskothen-Kuhl, 2012) and 1 day (d) (C) of unilateral stimulation *(us*). Fos-positive neurons were spatially limited to regions tonotopically corresponding to frequencies processed at the site of intracochlear electrode position. The number of labeled neurons was higher after 73 min than after 1d of *us*. Inset A: AVCNi was devoid of staining after incubation with Fos sense probe. Inset C: higher magnification of mRNA-positive neurons, showing strongest staining in cytoplasm. (B, D) Fos protein staining (black dots) in AVCNi after 73 min (B; cp. Illing and Rosskothen-Kuhl, 2012) and 1d (D) of *us*. Protein expression was spatially limited to a band tonotopically corresponding to intracochlear stimulation position. Inset B: AVCNi of control. Scale bars in insets A and B: 0.2 mm. Inset D: higher magnification of Fos protein positive nuclei in AVCNi. Scale bars in insets C and D: 20 μm. (E) Three days after sustained EIS a strong decrease in the number of Fos protein positive nuclei was observable. (F) Following 5d of *us,* no further protein positive nuclei were detectable. Scale bar for A to E: 0.2 mm. nVIII: 8th cranial nerve.

Fos mRNA and protein staining served as a marker for the effectiveness of tonotopic activation of auditory brainstem neurons. In controls, only a negligible number of Fos mRNA and protein positive neurons was seen anywhere in the auditory brainstem (cp. Fig. 9A, B, inset). Activated by chronic EIS showing four identifiable positive peaks in the EABR (Fig. 2C), neurons in the ipsilateral anteroventral cochlear nucleus (AVCNi) contained Fos mRNA and protein in a regionally restricted area. This region corresponded tonotopically to the intracochlear stimulation site (Fig. 9). A correspondingly restricted population of Fos positive neurons was seen in LSOi and CICc [39]. Following one day of sustained EIS, only a sparse level of Fos mRNA positive neurons was observed, while a slightly reduced but still recognizable number of neurons expressing Fos protein existed in these auditory regions (cp. Fig. 9C, D). By 3 days of chronic EIS, the number of neurons stained for Fos protein has even more decreased in the activated area (Fig. 9E). Following EIS for longer than 3 days, no mRNA and protein positive neurons were detected in VCN, LSO, and CIC (cp. Fig. 9F).

## Discussion

**Figure 10:**
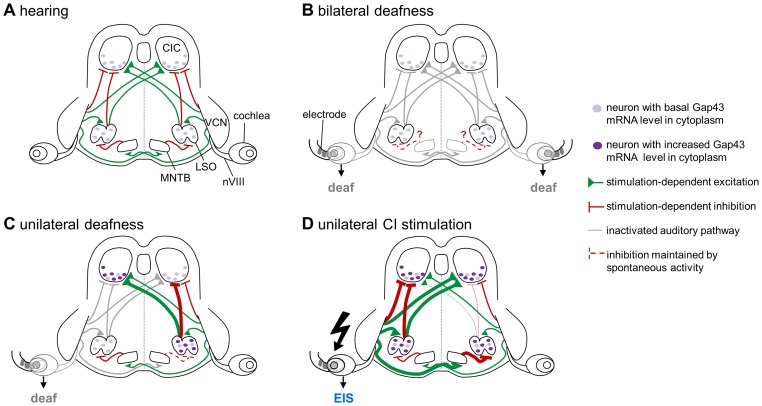
Expression level of Gap43 mRNA depends on synaptic cooperation in auditory brainstem nuclei. (A) Under control conditions, neurons in LSO and CIC of both sides of the brainstem receive stimulation-dependent excitatory (green) and inhibitory (red) inputs defining a functional balance with respect to metabotropic receptor activation to generate a basal level of Gap43 mRNA expression (light purple dots). (B) Induction of bilateral deafness silences all stimulation-dependent inputs (gray), leaving Gap43 mRNA levels unchanged. Question marks indicate a possible influence of spontaneous activity in MNTB neurons of unknown significance. (C) Unilateral deafness causes an imbalance of excitation and inhibition on neurons. Loss of excitation (gray) remained ineffective in modulating Gap43 mRNA levels in LSOi and CICc, whereas loss of stimulation-dependent inhibition for LSO via MNTB induces a significant increase of Gap43 mRNA staining level (dark purple dots) in LSOc and CICi. (D) Following unilateral EIS, excitatory afferents of neurons in LSOi and CICc as well as inhibitory afferents on neurons in LSOc and CICi were kept active even if the CI induced input is stronger-than-normal (thick lines). This resulted in a deviation from the normal excitatory-to-inhibitory input ratio and led to a rise of *gap43* transcription in neurons of these auditory regions (dark purple dots). nVII: 8th cranial nerve; MNTB: medial nucleus of the trapezoid body; LSO: lateral superior olive; CI: cochlear implant; CIC: central inferior colliculus; EIS: electrical intracochlear stimulation; VCN: ventral cochlear nucleus.

This study is the first to demonstrate that *gap43* transcription may be modulated by the level and pattern of sensory activity in an intact adult mammalian brain. Studies involving transgenic mice overexpressing *gap43*, *gap43* knock-down, or *gap43* gene silencing have collected an impressive body of evidence suggesting the protein Gap43 to be involved in axonal growth and the formation and plasticity of synaptic contacts [8]–[12]. Specifically, the neuronal transcription of *gap43* is changed asymmetrically in the central auditory brainstem as soon as sensory evoked activity is unilaterally lost. However, the formation of such asymmetry between both sides of the brainstem can be prevented. A simple-patterned stimulation of the auditory nerve on the side of a deaf ear is sufficient to maintain the bilateral balance of Gap43 mRNA on the levels of LSO and CIC (Fig. 10).

### Asymmetric Activation of the Adult Auditory System Triggers Imbalanced *gap43* Transcription

In a normally functioning adult rat brain and in brains of unilaterally or bilaterally hearing impaired rats, neurons of LSO and CIC maintain the same ipsilateral-to-contralateral ratios of *gap43* transcription close to 1. Based on this result, we pooled these groups as our statistical reference (Co; Figs. 6, 7). The lack of massive molecular changes by a rise of hearing threshold of 50 dB suggests that even a notably dampened auditory environment is sufficient to maintain a qualitatively normal pattern of neuronal activity due to aural and interaural stimulation. The level of this transcription remains unchanged by a bilateral loss of sensory input. However, total deafness of only one ear quickly results in an asymmetric change of *gap43* transcription when comparing both sides of the brainstem. One cause for this molecular imbalance must be the changed ratio of excitatory-to-inhibitory inputs of LSO and CIC neurons. While neurons in LSOi and CICc are released from their ascending excitatory input, neurons in LSOc and CICi lost their ascending inhibitory input [20], [29] (Fig. 10C).

However, despite a changed excitatory-to-inhibitory ratio in LSO and CIC under *ud*, Gap43 mRNA levels rose only on the side where stimulation-dependent inhibition was silenced. If LSOi of *ud* rats receives glycinergic inhibition from MNTBi but no longer excitation from VCNi, neurons maintain a basal level of *gap43* transcription. The same applies when CICc is no longer excited by a crossed innervation from VCNi and LSOi, but inhibitory afferents from LSOc [40], [41] are still active (Fig. 10C). By contrast, if LSOc lost its stimulation-dependent inhibition by way of MNTBc, an increase of Gap43 mRNA results (Fig. 10C). The same applies if CICi is excited by the sensory activated VCNc across the midline, but now misses inhibition from silenced LSOi and VCNi as a consequence of deafness [29], [42]. Remarkably, this deafness-dependent induction of bilaterally asymmetric expression of Gap43 mRNA appeared to be stable. Even an auditory system unilaterally deafened for a longer time failed to recover the initial Gap43 mRNA symmetry by an intrinsic regime (cp. Fig. 5E, F), a finding matching earlier observations made after unilateral cochlear ablation [18].

It is important to note that spiking activity cannot be the sole cause for *gap43* transcription or its modulation. If it were, we should have seen some change in Gap43 mRNA expression of VCN neurons which we never did. This evidence is the first to suggest that neuronal activity plays upon *gap43* transcription in a fundamentally different way than upon *fos* transcription.

### Unilateral EIS Prevents Asymmetric *gap43* Transcription in the Auditory Brainstem

When one ear of hearing-experienced adult rats was supplied with a CI and electrically stimulated while the other continued transduction of acoustic signals, a tonotopic expression of neurons containing Fos mRNA or protein was seen only in auditory regions of the electrically activated pathway (Fig. 9 [39]). This observation implies that both sides of the auditory brainstem received different strengths and patterns of sensory-evoked input. Remarkably, the same tonotopically restricted simple-patterned CI stimulation maintained the symmetry of *gap43* transcription in LSO and CIC on both sides of the brainstem rather than letting it drift into bilateral imbalance (Fig. 10D). Apparently, CI stimulation can remedy molecular consequences of a failing ear by giving auditory brainstem circuits an input sufficient to maintain synaptic cooperation on both sides of the brainstem. However, the expression level of Gap43 mRNA is significantly increased on both sides of the brainstem under this specific condition of mixed bilateral activation. An increase of Gap43 mRNA expression can also be induced by nerve crush of the facial nerve followed by retrograde electrical stimulation of its nucleus in the adult mammalian brain [43].

Given the presence of Gap43 mRNA and the moldability of its quantity, we propose that the molecular machinery for neural circuit adaptation and reorganization is maintained in the adult auditory brainstem waiting to be unlocked. Our study shows that variations in strength and pattern of sensory input have significant impact on the expression of plasticity-related genes, such as that encoding for Gap43. We demonstrated that levels of Gap43 mRNA expression reflect altered sensory activity in conjunction with a shifted ratio between excitation and inhibition on neurons of the auditory brainstem.

### Rules Governing the Modulation of *gap43* Transcription

Considering the modulation of the basal level of *gap43* transcription under various experimental conditions, we propose the following rule: Gap43 mRNA expression rises above baseline level in neurons whenever the ratio of their excitatory-to-inhibitory input changes, but only as long as both types of synapses are active (Fig. 10).

In case of bilateral hearing loss (*bd)*, neurons of LSO and CIC lost both excitatory and inhibitory synaptic inputs, failing to detect a deviation from their standard ratio. As a result, neurons maintained *gap43* transcription as normal (Fig. 10A, B).

When sensory input failed on one side only (*ud*), a sustained basal *gap43* transcription persisted in neurons of LSOi and CICc, since they lost their excitatory input (Fig. 10C). By contrast, neurons in LSOc increased *gap43* transcription since stimulation-dependent inhibition by MNTBc failed due to inactivation of driving neurons in VCNi, while excitation from VCNc remained effective. As neurons of MNTB maintain a moderate spontaneous activity [44], [45], glycinergic presynaptic endings on LSOc neurons are still effective in supporting second messenger systems. It is unknown if this spontaneous activity is maintained in a totally deaf brain (Fig. 10B, question marks). Thus, LSOc neurons are more strongly excited than normally but are still affected by glycinergic synapses so that second messenger cascades triggered from both glutamatergic and glycinergic receptors can interact intracellularly and increase Gap43 mRNA expression.

This dependence of *gap43* transcription on synaptic cooperation marks a sharp contrast to the regulation of *fos* expression apparently characterizing neurons exclusively or predominantly affected by excitatory input. From all 16 sites investigated for their Gap43 mRNA expression (LSO and CIC bilaterally under 4 experimental conditions, cp. Fig. 10), CICi under *ud* is the one not readily explained by the rule suggested above. As CICi under *ud* should lose inhibition by LSOi and VCNi but maintain strong excitation supplied by LSOc and VCNc, we need to additionally postulate that a disinhibition of its neurons may be sufficient to directly increase the *gap43* transcription (Fig. 10C).

Compensating a dysfunctional ear by EIS (*us*) increased sensory activity in an otherwise deaf ear. As a consequence, LSOi and CICc received a stronger-than-normal excitatory synaptic input, and inhibition is increased for LSOc and CICi at the same time. This resulted in a deviation from the normal excitatory-to-inhibitory input ratio and led to a rise of *gap43* transcription in neurons of these auditory regions (Fig. 10D).

### Comparison of Gap43 mRNA and Protein Levels in the Auditory Brainstem

An important question concerns the unlocking of a neuron’s potential to grow as indicated by the intracellular presence of Gap43 mRNA. Translation into protein does not trivially follow from mRNA expression. In our study, basal levels of Gap43 protein known to be quickly transported from the neuronal soma to its nerve terminals [46] were observed independent of experimental treatment (Fig. 8B–D).

### VCN

By postnatal day 28, *gap43* transcription almost completely disappears from the rat VCN [18]. Correspondingly, we detected no Gap43 mRNA positive neurons in VCN of adult rats (Fig. 8A). Since this remained unchanged under any experimental condition, we suggest that VCN neurons lack the competence for initiating growth responses in adulthood. However, a basal level of Gap43 protein always existed in presynaptic endings throughout VCN (Fig. 8B, and arrowheads in inset). Since Gap43 mRNA was absent from VCN, the source of the Gap43 protein has to be elsewhere. A plausible candidate is the superior olivary complex. Cholinergic medial olivocochlear neurons of the ventral nucleus of the trapezoid body are known to be a major source for Gap43 protein in VCN [37], [38], [47]. Additionally, small neurons of the LSO of which some were calbindin positive [48], [49], and shell neurons of the periolivary region around the LSO maintain axons heading for VCN and are potential parent cell bodies of presynaptic endings found there [48].

### LSO

While the deviation of Gap43 mRNA expression from baseline level followed from changes of the ratio of excitatory-to-inhibitory synaptic input driven from both ears in LSO (Fig. 6A, B), levels of Gap43 protein failed to do so. Instead, only selected LSO neurons expressed this protein on the side of deafness-inducing electrode implantation by 5 days, independent of EIS (Fig. 8C, arrows and inset). Following cochlear ablation in adult rats, such neurons emerge throughout LSOi by 5 to 7 days and were identified as lateral olivocochlear cells [19]. We demonstrated before [38] that these neurons do not have the ability to sprout into VCN after cochlear ablation.

### CIC

Ventral neurons within CIC changed their *gap43* transcription as a function of balance and strength of binaural activity. Simultaneously, numerous presynaptic endings positive for Gap43 protein were found throughout CIC and remained unaltered under different experimental conditions (Fig. 8D, and inset). Complying with physiological studies investigating interactions of bilateral acoustic stimulation in IC [31], our observations reinforce the concept of IC neurons maintaining a potential for experience-dependent adjustments even at adult age.

Overall, in the system studied here, no obvious quantitative relationship between the presence of Gap43 mRNA and protein was detected. It seems plausible, though, to suspect some functional reason behind energy and logistics expenses required for maintaining Gap43 mRNA expression in specific neuronal populations of the adult auditory brainstem. Neurons containing Gap43 mRNA in LSO and CIC may be waiting and ready for neuroplastic growth that might require another yet unidentified ‘go’ signal. It would be extremely important to identify the nature of this signal for understanding adult brain plasticity in general, and to put it to use for therapeutic approaches of CI patients in particular.

### *Gap43* Transcription does not Trivially Depend on Fos Expression

Looking for the upstream regulation of *gap43* expression, the transcription factor AP-1 and Fos as one of its monomers are obvious candidates [4], [50]. The results of the present study unequivocally indicate that the transcription of *gap43* does not trivially depend on the availability of Fos. Stimulating the auditory system by unilateral EIS, Fos mRNA and protein expression increased in VCNi, LSOi, or CICc [34], [39], [51]–[53] (Fig. 9), among others. In *ud* rats, no increase of Fos expression was detectable in regions of the auditory pathway on the opposite side, but neurons of LSOc and CICi significantly increased their Gap43 mRNA transcription.

Looking closer, a local activation of the medial turn of the cochlea by EIS resulted in a tonotopically restricted Fos expression within most central auditory regions [2], [39] (Fig. 9). However, a matching local restriction was not recognizable in the *gap43* transcription pattern of LSO or CIC under identical stimulation conditions. As mentioned above, VCN prominently expressing Fos after 1 or 3 days of chronic EIS never showed Gap43 mRNA positive neurons. For these reasons, no evidence can be cited to assume a dominant role of Fos in the regulation of *gap43* transcription. Apparently, then, regulation of *gap43* expression must be under the control of additional or other intracellular signaling pathways.

### Clinical Relevance

In adult humans, monaural deafness can be a result of acute hearing loss, cochlear ossification, or acoustic neuroma surgery. Different medical response modalities exist for these patients, ranging from ‘no treatment’ up to providing the deaf ear with one of various types of hearing aids [54]. Recent studies demonstrate that EIS by way of a CI improves hearing abilities in patients with single-sided deafness and is superior to alternative treatment options mostly relying on monaural hearing alone [54], [55]. Binaural hearing is more demanding on network functions and must have cellular and molecular correlates. We propose that our findings of molecular changes in auditory neurons due to variations in the strength and pattern of sensory input provide important details about these network dynamics. A major outcome of our study is that even a simple-patterned CI stimulation may remedy molecular consequences of a failing ear in central auditory neurons.

### Conclusion

As suggested by Moore and Shannon [56], a deeper understanding of central auditory plasticity responses is required to further improve the effectiveness of CI-brain-interactions in patients. Proceeding in this direction, our study identifies a regulating mechanism including the transcription of the plasticity and growth associated gene *gap43* within the adult auditory brainstem. From these data we derive the hypothesis that the level of *gap43* transcription rises when two conditions are met: (1) the ratio of excitation to inhibition must deviate from normal, and (2) both types of synapses, excitatory and inhibitory, must be active to invoke second messenger cascades to interact intracellularly. In our series of experiments, these conditions are met if one ear is completely deafened or else permanently activated by EIS. With this study we provide new insights into the readiness of neurons in the adult brainstem to initiate axonal growth. The next step must be to find out how to unlock this potential.
